# Screening for Mutations in Isolated Central Hypothyroidism Reveals a Novel Mutation in Insulin Receptor Substrate 4 

**DOI:** 10.3389/fendo.2021.658137

**Published:** 2021-05-21

**Authors:** Konrad Patyra, Kristiina Makkonen, Maria Haanpää, Sinikka Karppinen, Liisa Viikari, Jorma Toppari, Mary Pat Reeve, Jukka Kero

**Affiliations:** ^1^ Research Centre for Integrative Physiology and Pharmacology, Institute of Biomedicine, University of Turku, Turku, Finland; ^2^ Turku Center for Disease Modeling, University of Turku, Turku, Finland; ^3^ Department of Genomics and Clinical Genetics, Turku University Hospital, Turku, Finland; ^4^ Department of Genetics, University of Turku, Turku, Finland; ^5^ Department of Pediatrics, Turku University Hospital, Turku, Finland; ^6^ Institute for Molecular Medicine Finland, HiLIFE, University of Helsinki, Helsinki, Finland

**Keywords:** insulin receptor substrate 4, IRS4, central hypothyroidism, thyroid disorders, genetic screening, FinnGen, congenital hypothyroidism

## Abstract

**Background:**

Central hypothyroidism (CeH) is a rare condition affecting approximately 1:16 000- 100 000 individuals. Congenital forms can harm normal development if not detected and treated promptly. Clinical and biochemical diagnosis, especially of isolated CeH, can be challenging. Cases are not usually detected in neonatal screening, which, in most countries, is focused on detection of the more prevalent primary hypothyroidism. Until now, five genetic causes for isolated CeH have been identified. Here we aimed to identify the genetic cause in two brothers with impaired growth diagnosed with CeH at the age of 5 years. We further evaluated the candidate gene variants in a large genetic database.

**Methods:**

Clinical and biochemical characterization together with targeted next-generation sequencing (NGS) was used to identify the genetic cause in a family of two brothers presenting with CeH. Screening of *insulin receptor substrate 4* (*IRS4*) variants was carried out in the FinnGen database.

**Results:**

A novel monoallelic frameshift mutation c.1712_1713insT, p.Gly572Trp fs*32 in the X-linked *IRS4* gene was identified by NGS analysis in both affected males and confirmed using Sanger sequencing. Their mother was an unaffected carrier. In addition to the declined growth at presentation, central hypothyroidism and blunted TRH test, no other phenotypic alterations were found. Diagnostic tests included head MRI, thyroid imaging, bone age, and laboratory tests for thyroid autoantibodies, glucose, insulin and glycosylated hemoglobin levels. Examination of the *IRS4* locus in FinnGen (R5) database revealed the strongest associations to a rare Finnish haplotype associated with thyroid disorders (p = 1.3e-7) and hypothyroidism (p = 8.3e-7).

**Conclusions:**

Here, we identified a novel frameshift mutation in an X-linked *IRS4* gene in two brothers with isolated CeH. Furthermore, we demonstrate an association of *IRS4* gene locus to a general thyroid disease risk in the FinnGen database. Our findings confirm the role of *IRS4* in isolated central hypothyroidism.

## Introduction

Central hypothyroidism (CeH) is defined as a reduced thyroid hormone secretion from an otherwise functional thyroid gland due to diminished stimulation of the gland. Reduced stimulation can result either from an impaired secretion of thyroid stimulating hormone (TSH) from the anterior pituitary, defective secretion or action of hypothalamic thyrotropin releasing hormone (TRH), or both (1).

Central hypothyroidism is rarely an isolated defect, but most often appears congenitally as a part of panhypopituitarism affecting also gonadotropin, adrenocorticotrophic hormone (ACTH) or growth hormone secretion. Panhypopituitarism can be potentially life-threatening, primarily because of severe hypoglycemia. The exact prevalence of CeH is unknown, but the presentation of genetic forms shows a peak in childhood, whereas other forms due to pituitary lesions are typical later in adulthood (2). Estimations of CeH prevalence vary between 1:16 000-100 000 individuals (3, 4). Overall, CeH has been reported to be equally distributed in both sexes. However, known X-linked forms suggest a male predominance (2). In addition to the genetic etiology of isolated central hypothyroidism, impairing only TSH secretion, CeH has been described in patients with pituitary tumors, trauma, radiation therapy, diabetes mellitus or it may be idiopathic (5). Genetic defects in pituitary transcription factors can lead to CeH, but are usually associated also with other hormonal defects (1). Isolated TSH deficiency covers approximately 20% of all CeH cases (2). Inherited isolated CeH has been demonstrated to be caused by mutations in *thyroid releasing hormone receptor* (*TRHR*), *thyroid stimulating hormone beta subunit* (*TSHB*), *immunoglobulin superfamily member 1* (*IGSF1*), *transducin (beta)-like 1X-linked* (*TBL1X*) and more recently, in *insulin receptor substrate 4* (*IRS4*) genes (6). Among these genes *TRHR* mutations has been shown to lead to blunted TSH response to TRH, growth retardation and obesity during childhood (7). Furthermore, *TSHB* mutations are characterized by neonatal onset of low TSH, high glycoprotein hormone alpha-subunit levels and pituitary hyperplasia, which is reversible with thyroxine treatment (8). Mutations in both *IGSF1* and *TBL1X* can lead to X-linked isolated CeH, but *IGSF*1 mutations are also associated with low PRL, variable GH deficiency, metabolic syndrome, and postpubertal macroorchidism (9). In addition, the mutations in the *TBL1X* can also lead to impaired hearing (10).

Here we describe a genetic, biochemical and clinical characterization of a family with two brothers diagnosed with CeH at the age of 5 years and shown to carry a novel frameshift mutation in the *IRS4* gene. Furthermore, we evaluate the overall occurrence of *IRS4* variants and their association to other clinical phenotypes in a large national genetic database.

## Materials and Methods

### Study Participants

The study participants were recruited to the study by a pediatric endocrinologist, and they and their parents signed a written consent. The Ethics Committee of the Hospital District of Southwest Finland approved the study (108/180/2010).

The clinical examinations were performed by a pediatrician (SK) and thyroid ultrasound, head MRI and bone age by the pediatric radiologists. Laboratory tests were done at the Turku University Hospital Laboratory, except for the IGF-1 test, which was performed in the Islab-laboratory (Kuopio, Finland). Umbilical serum TSH (uS-TSH), serum TSH, free T4 (fT4), insulin and cortisol concentrations were determined with the Cobas e801 immunoassay analyzer (Roche Diagnostics, Rotkreuz, Switzerland). Serum cholesterol (HDL cholesterol, LDL cholesterol and triglycerides) were determined with the Cobas c702 chemistry analyzer (Roche Diagnostics). Serum glycosylated hemoglobin (HbA1c) was determined with the Cobas c501 immunoturbidimetric assay (Roche Diagnostics). Serum IGF-1 concentrations were determined with the Liaison XL chemiluminescence analyzer (DiaSorin S.p.A, Saluggia, Italy). Growth data were collected from the hospital records based on measurements performed at the visits using stabilized and calibrated scale and wall mounted Harpenden Stadiometer (Holtain Limited, Crosswell, Crymych, Pembs., UK) with ±0.1 cm precision.

### TRH Stimulation Test

At the start of the TRH stimulation test, non-fasting serum TSH concentrations were measured. A bolus of 7 μg/kg of TRH (Ferring Pharmaceuticals, Saint-Prex, Switzerland) was given intravenously, and subsequently the serum TSH concentrations were measured at 20 and 60 minutes.

### Genetic Analysis

Genetic analyses were performed on DNA extracted from peripheral blood. Amplification of target region was performed with PCR using AmpliTaq Gold 360 (ThermoFisher Scientific, Waltham, MA, USA) according to the manufacturer’s protocol in the Veriti 96-Well Thermal Cycler (Applied Biosystems, Foster City, CA, USA). Primers used are listed in the **Supplemental Table 2**. For the index patient an NGS-based targeted panel was performed with Sophia Genetics custom clinical exome solution (Sophia Genetics, Boston, MA, USA) and Illumina sequencing (Illumina, San Diego, CA, USA), including 4400 known disease-causing genes. NGS libraries were prepared using a hybrid capture method according to the manufacturer´s protocol (Sophia Genetics CCE_A_v1). DNA was sequenced with NextSeq sequencer (Illumina, San Diego, CA, USA) using 2x151bp paired-end technique. The identified variation was visually inspected using the Integrative Genomics Viewer (11). Bioinformatic analysis and annotation was focused on the following candidate gene panel for CeH: *HESX1, IGSF1, IRS4, LEPR, LHX3, LHX4, OTX2, POU1F1, PROP1, SOX3, TBL1X, TRHR* and *TSHB*.

Confirmation of the IRS4 mutation and its segregation was tested with PCR and Sanger sequencing. The primer sequences and PCR conditions are listed in the **Supplementary Materials**. Sequencing reactions were performed by using BigDye Terminator v3.1 Cycle Sequencing Kit (Applied Biosystems). Sequencing was performed with the ABI3500xl Dx (Applied Biosystems) and chromatograms were analyzed using Sequencher v5. (Gene Codes Corporation, MI, USA). The alignment of WT and mutated IRS4 sequences was performed using the Clustal O (1.2.4) multiple sequence alignment tool (12).

### IRS4 Variant Analysis in the FinnGen Database

The FinnGen project has been approved by the Ethical Review Board of the Hospital District of Helsinki and Uusimaa with the protocol Nr. HUS/990/2017. The FinnGen data release 5 was used. Detailed information about of the different releases is described on the FinnGen’s website^1^. Release 5 comprises of data from 218 792 Finnish participants with disease endpoints^2^ constructed from national registries using International Classification of Diseases (ICD), Social Insurance Institute (KELA) drug reimbursement and ATC codes linked with DNA data.

## Results

### Clinical Characteristics

**Figure 1:**
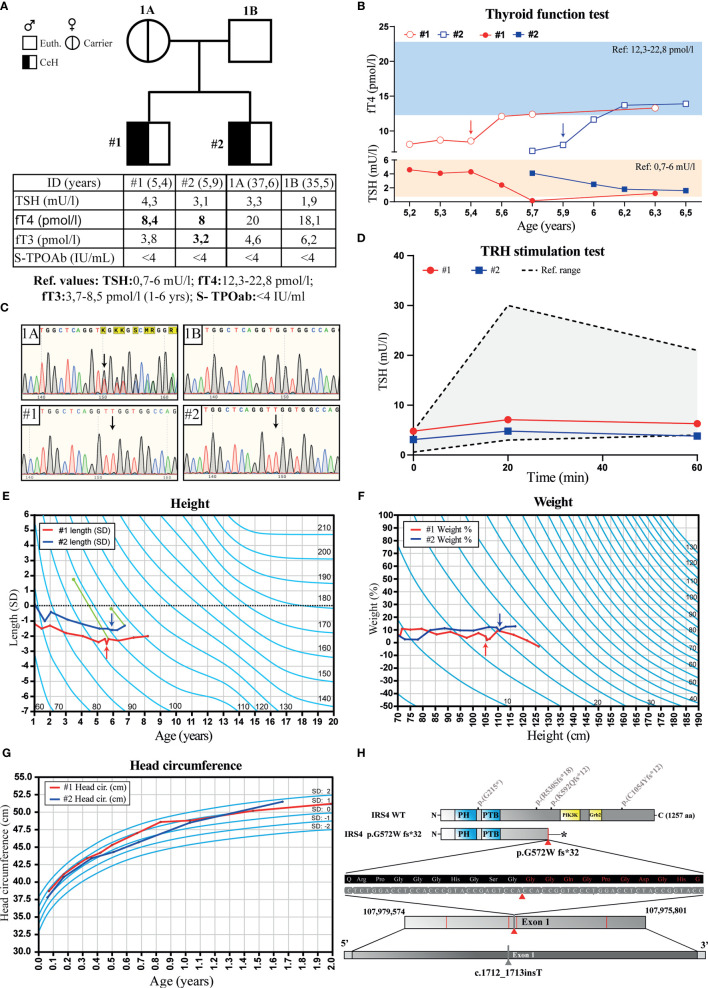
Pedigree, thyroid function tests, growth charts and chromatograms of the family with two brothers diagnosed with isolated central hypothyroidism (CeH). **(A)** Pedigree, serum TSH, fT4, fT3 and TPO antibody (S-TPOAb) concentrations in parents and their offspring. Bolded values indicate concentration below the reference range. **(B)** Follow-up graph of the serum TSH and fT4 concentrations in the affected cases before and after the thyroxin replacement therapy. Blue and orange rectangles show fT4 and TSH reference ranges, respectively. **(C)** Chromatograms of the IRS4 sequence flanking the mutation in mother (1A), father (1B), and the two patients #1 and #2 presenting CeH. Black arrows show the position of thymidine insertion. **(D)** TSH response in TRH stimulation test of the affected cases, gray area indicates a range of normal TSH response. **(E)** Growth (height SD) and **(F)** weight (%) curves of the affected siblings: #1 (red) and #2 (blue). Arrows indicate the time of CeH diagnosis and the start of thyroxine treatment. The green dots and lines show bone age determined at the indicated calendar age using the Tanner-Whitehouse method, and dotted line shows the expected length calculated from the parents´ heights. **(G)** Head circumference in affected siblings carrying IRS4 mutation during the first 2 years. Light blue lines show SD values. **(H)** Location of the IRS4 frameshift mutation detected in this study (marked with red triangle), premature stop codon (indicated with star) previously published (6) *IRS4* mutations (marked with red lines). The location of IRS4 gene shown is based on the GRCh37.p13 primary assembly.

The two male subjects were referred to the endocrinologist due to mild growth retardation and normal TSH, but low serum fT4 concentrations. They were both diagnosed with CeH between the age of 5 – 6 years. The index case (patient #1) and his brother (patient #2) had normal serum TSH and low fT4 values prior the diagnosis (**Figures 1A, B**). Both cases had blunted TSH response (ΔTSH, <1.6) to TRH stimulation (**Figure 1D**), no interfering antibodies in TSH or TH assays, and the TG or TPO antibody tests were negative (**Figure 1A**, table). Secretion defects or dysfunction of other pituitary hormones were excluded (**Supplemental Table 1**). Both MRI of the head and ultrasound evaluation of the thyroid were normal in both cases at the time of diagnosis. Patient #1 had linear growth until the age of 2 years, after which his height standard deviation scores (SDS) decreased from –1 to –2.4 SD between the age 2 and 5 years (**Figure 1E**). His brother (#2) had similar growth retardation prior to the CeH diagnosis. Both had delayed bone age (2.0 - 2.3 and 0.7 years behind the calendar age) at the time of diagnosis. There were no significant alterations in other biochemical or metabolic variables measured including glucose, glycosylated hemoglobin (HbA1c), insulin, insulin-like growth factor-1, cholesterol or cortisol levels (**Supplemental Table 1**). The thyroxine replacement was started promptly after CeH diagnosis and fT4 levels returned to normal. Furthermore, the serum TSH concentrations, although within the normal range at diagnosis, decreased significantly in both cases after thyroxine supplementation. A small increase in growth velocity was also noted after the initiation of thyroxine treatment (**Figure 1E**).

Both brothers were born at term after a normal pregnancy and uncomplicated birth with >9 APGAR-scores. However, patient #1 received phototherapy for prolonged jaundice as a newborn. The birth weight, height and head circumferences were within average Finnish standards. Patients #1 and #2 had normal umbilical serum TSH levels (patient #1: 11 mU/l and patient #2: 6.6 mU/l) measured at birth as a part of the CH screening program. Both brothers reached all developmental milestones, had normal weight gain and head growth pattern during the first two years (**Figures 1F, G**), normal weight gain and development and no further diagnoses. Both parents were healthy, had thyroid function tests (TSH, fT4, fT3) in normal range, and negative TPO and Tg antibody tests, when measured at the recruitment visit. There was no positive family history for thyroid disease.

### Genetic Findings

A novel frameshift variant was detected in hemizygous state in exon 1 of the *IRS4* gene located on the X chromosome (X:107977861; NM_003604.2) of the affected male proband (**Figure 1H**). The detected variant had a 1bp insertion (c.1712_1713insT), which leads to a frameshift mutation (p.Gly572Trp fs*32, amino acid sequence listed in **Supplemental Figure 1**), premature stop codon and strongly truncated protein (**Figure 1H**). This variant most likely degrades through nonsense-mediated decay and is not present in the gnomAD and dbSNP databases. Confirmation and co-segregation of the mutant in the family was performed using Sanger sequencing. The same variant was also found in the DNA of the affected brother and the mother, who was a healthy carrier (**Figures 1A, C**). No other pathogenic variants associated with CeH were detected in the clinical exome.

### Exploring IRS4 Mutations in the FinnGen Study

To explore the role of IRS4 in both hypothyroidism and across the medical spectrum, we used the large FinnGen population study which in release 5 had integrated genome-wide genotyping and extensive medical history data from 218 792 Finns. We searched for any rare loss-of-function (LoF) or damaging missense mutations (**Table 1**) and found no LoFs. Of note, the only rare predicted damaging missense variant observed (rs766893547, p.Arg8His) was carried by two female congenital hypothyroidism cases (an enrichment odds ratio of 6.7 compared to all females, p = 0.03). Furthermore, another variant (rs1801164) had significant association to renal failure (**Supplemental Figure 2**).

**Table 1:** IRS4 missense variants in the FinnGen-database.

Location	source	rsid	HGVSp	poly-phen	N(m/f)	fin.AF	nfsee.AF
23:108733710:G:C	I	rs1801164	p.His879Tyr	U	**ND**	0.14554	0.2104144
23:108734099:C:T	C	rs774511400	p.Arg749Lys	U	32/124	0.00049966	0
23:108735030:T:A	I	rs137853896	p.Ser439Cys	B	ND	0.0063782	0.00043206
23:108735113:C:T	I	rs41307415	p.Arg411Gln	B	ND	0.030372	0.05821377
23:108736245:G:A	I	rs1801162	p.Leu34Phe	B	ND	0.030347	0.05845302
23:108736257:C:A	C	rs769861641	p.Val30Leu	B	12/75	0.00025102	0
23:108736322:C:T	C	rs766893547	p.Arg8His	PB	25/110	0.00031291	0

IRS4 Missense variants in the FinnGen-database. Variant source; I (imputed) or C (variant detected from chip); rsid. rs number; HGVSp, the HGVS protein sequence name (genome build 38); Polyphen: U, unknown, B, benign; PB, probably damaging; N, number of males (m) and females (f); ND, not detected; fin.AF, allelic frequency in Finnish; nfsee, non-Finnish-non-Swedish-non-Estonian European.

We then surveyed the IRS4 locus to see if any medical phenotypes might be associated to this genomic region. This was done by evaluating the DNA variants at the IRS4 locus in FinnGen participants and comparing those to disease phenotypes^2^ obtained from national registries linked with the DNA data. Across all 2925 phenotypes studied in the FinnGen project, the strongest associations discovered were with thyroid disorders (p = 1.3x10-7) and hypothyroidism (p = 8.3x10-7), which were associated to a rare Finnish-enriched haplotype (tagged by SNP rs1452561670 20 kb downstream of IRS4) (**Supplemental Figure 2**). This haplotype spans a roughly 500 kb interval within which IRS4 is the only documented protein-coding gene.

## Discussion

Isolated central hypothyroidism (CeH) in children is rare and challenging to diagnose clinically. It has a multifactorial etiology and a small proportion of cases that are due to gene mutations. Therefore, the etiology for several cases remains unsolved. Currently, mutations in five different genes have been shown to associate with CeH. In this study, we describe a genetic, clinical and biochemical characterization of a family with two brothers presenting CeH at the age of 5 years. Furthermore, an evaluation of candidate gene variants for isolated CeH and their putative association to other phenotypes in a national genetic database was performed. In the present study, both affected cases with a novel frameshift mutation in an X-linked *IRS4* gene were shown to have normal umbilical TSH measured at newborn screening and normal psychomotor development. However, their growth started to slowly decline 2-3 years prior to the diagnosis of CeH, which prompted the investigations. The TSH response was blunted in the TRH-test and TSH levels declined from mid-normal to low- normal after start of the thyroxine replacement. The mother was a healthy carrier with normal TSH and also fT4 concentrations at the upper normal range. The data from this family supports the pathogenic role of IRS4 in isolated central hypothyroidism and the cases showed no obvious additional phenotypes. Furthermore, the candidate gene analysis using the FinnGen database showed an association between the *IRS4* locus and thyroxine purchases.

So far, only one study has reported the association of CeH with pathogenic mutations in *IRS4* (6). Heinen et al. described five families and seven affected male patients with four different mutations in the *IRS4* gene. Most of those patients were detected at birth *via* neonatal screening which can identify both primary and CeH (13). Unfortunately, CeH is not usually detected in congenital hypothyroidism (CH) screening programmes, which are generally targeted to detect the more prevalent primary CH using TSH-based screening (4, 14). However, in some countries with T4-based screening programmes CeH can be detected at birth and potential neurodevelopmental disabilities can be therefore prevented. The importance of such screening was recently demonstrated in a study by Lanting et al. (15), which showed a relatively high (1 in 16 404) occurrence of CeH. Moreover, this finding is also supported by the fact that the clinical recognition of isolated CeH can be challenging without the classic symptoms, such as hypoglycemia, jaundice and micropenis observed typically in panhypothyroidism (2). Thus the debate continues whether the screening should also detect central CH continues (15, 16). In Finland, TSH-based screening is used and the CeH cases have not been routinely detected (17). The two cases described in our study, show that the impaired IRS4 function does not necessary lead to severe CH, as the boys´ development, growth and head growth for the first 2 years was normal. Similarly, in the study from Heinen et al. (6) they report a male case diagnosed with CeH at the age of 12, originally evaluated because of short stature and delayed tooth eruption but otherwise normal development. This suggests that a significant degree of compensation can occur either in the regulation of the growth during the infancy and childhood phase or at the thyroid axis level. In fact, the fT3 levels in our patients were normal or only slightly below the reference range, and normal among the patients described in the previous study (6). In the previously described *IRS4* mutation cases the TSH response to the TRH-test was blunted in six of seven male *IRS4* mutation carriers, similar to that seen in our patients. The value of the TRH-test in the diagnosis of CeH with multiple etiologies has been shown to be controversial (18, 19), and the response can be affected by multiple factors such as initial TSH level, age, weight or nutritional state (20–22). However, the blunted TSH response seen in our CeH cases and the previously described *IRS4* mutation carriers suggest that the IRS4 is needed for the proper signaling of the TRH. The *IRS4* gene codes for a cytoplasmic protein which interacts with tyrosine kinase receptors and mediates their signaling (23). It has been shown to be expressed in the hypothalamus, but also has been found in several other tissues including the pituitary, thyroid and ovaries (10). IRS4 knock-out mice exhibit mild metabolic differences including lower blood glucose levels, impaired glucose tolerance and decreased fertility (24). Additionally, IRS4 female knockout mice have decreased *Tshb* mRNA expression in the pituitary, but no altered serum TSH or thyroid hormone concentrations. In contrast, the lack of IRS4 function in humans impairs TSH pulsatile secretion but the detailed mechanisms remain unclear. Impaired TSH pulsatile secretion may be linked to leptin action (6) that has been shown to participate in TRH response (25) and its action is partially mediated *via* IRS proteins.

A limitation of our study is the lack of functional test of the mutation pathogenicity. However, *IRS4* mutation segregation in the family, the frameshift alteration leading to premature stop codon strongly support the pathogenicity of the mutation. Since the fine regulation of thyroid function *via* hypothalamus and pituitary seems to differ between human and mice (6) further functional studies, for example using induced human pluripotent pituitary cell lines, are warranted to elucidate the detailed mechanisms.

Using FinnGen data we could not identify new LoF mutations. However, one rare predicted damaging missense variant (rs766893547, p.Arg8His) was present in two females with a congenital hypothyroidism diagnosis. Furthermore, an association of the variant rs1452561670 to the thyroid endpoint category was observed. Although these associations include cases with central isolated hypothyroidism (ICD code E23.05), they may also indicate primary thyroid diseases.

To our knowledge, general association of the *IRS4* variants to thyroid diseases has not been previously described in GWAS studies or genetic databases. However, our observation of a Finnish-enriched haplotype spanning IRS4 would not be detectable outside of a large Finnish study. This haplotype spans a roughly 500 kb interval within which IRS4 is the only documented protein-coding gene.

In addition to the association to thyroid endpoint category in FinnGen one IRS4 missense variant (rs1801164) had a significant association to renal failure. However, no renal phenotypes were described in humans with *IRS4* frameshift mutations in the previous publication (6). Furthermore, in our study the creatinine values were normal and there was no sign of kidney dysfunction in the individuals with IRS4 mutation and the family history was negative for any kidney diseases. Therefore, the present clinical findings do not support this database association at least in young individuals.

In summary, our data from patients with a novel frameshift mutation in *IRS4* gene together with the observed association between the rare *IRS4* haplotype and thyroid disease risk supports the pathogenic role of IRS4 in isolated central hypothyroidism.

## Data Availability Statement

The original contributions presented in the study are publicly available. This data can be found here: https://www.ebi.ac.uk/eva/, PRJEB45041.

## Ethics Statement

The studies involving human participants were reviewed and approved by The Ethics Committee of the Hospital District of Southwest Finland (108/180/2010). Written informed consent to participate in this study was provided by the participants’ legal guardian/next of kin.

## Author Contributions

All authors listed have made a substantial, direct and intellectual contribution to the work, and approved it for publication.

## Funding

The study was supported by the Finnish Cultural and Sigrid Juselius (JK) Foundation (KP), Finnish Pediatric Foundation (JK) and a grant from Turku University graduate school (KM).

## Conflict of Interest

The authors declare that the research was conducted in the absence of any commercial or financial relationships that could be construed as a potential conflict of interest.

## Footnotes

1
https://finngen.gitbook.io/documentation/

2
https://www.finngen.fi/en/researchers/clinical-endpoints
