# Supplementary material for: Screening for Mutations in Isolated Central Hypothyroidism Reveals a Novel Mutation in Insulin Receptor Substrate 4
Source: Front Endocrinol (Lausanne). 2021 May 21;12:658137. doi: 10.3389/fendo.2021.658137 (PMC8176851; doi:10.3389/fendo.2021.658137)
Supplement: Supplementary file 1 [file DataSheet_1.pdf]

Supplementary Material

Supplemental table 1

Test	Patient #1	Patient #2	Reference
Umbilical serum TSH (mU/l)	11	6.6	< 40
Total Cholesterol (mmol/l)	5.0	4.5	2.7 - 5.8
HDL-Cholesterol (mmol/l)	1.71	1.23	0.93 - 1.94
LDL-Cholesterol (mmol/l)	3.4	3.2	1.6 - 3.6
Triglycerides (mmol/l)	0.6	0.8	< 1
Glucose (mmol/l)	4.4	5.0	4 - 6
HbA1c (mmol/mol)	33	35	20 - 42
Insulin (mU/l)	5	6	2.6 - 25
IGF1 (nmol/l)	27	23	5 - 44
Cortisol (nmol/l)	134	167	133 - 537
Bone age	2 – 2.3 years behind	0.7 year behind	
Head MRI	Normal	Normal	
Thyroid ultrasound	Normal (Isthmus 1,6 mm, left lobe 22 x 8 x 6 mm, right lobe 24 x 8 x 7 mm)	Normal (Isthmus 0,7 mm, left lobe 25 x 9 x 7mm, right lobe 30 x 9 x 7 mm)	

Clinical and biochemical variables and their reference values in two patients with CeH. Patient #1's laboratory tests were performed at the age of 9. Bone age was assessed at the age of 5.7 years. Patients #2's laboratory tests were performed at the age of 6.5 years and his bone age was determined at the age of 6.7 years.

```
WT_IRS4                       MASCSFTRDQATRRLRGAAAAAAAAALAAVVTTPLLSSGTPTALIGTGSSCPGAMWLSTAT      60
MUT_IRS4(p.G572W_fs*32)       MASCSFTRDQATRRLRGAAAAAAAAALAAVVTTPLLSSGTPTALIGTGSSCPGAMWLSTAT      60
                              ************************************************************

WT_IRS4                       GSRSDSESEEEDLPVGEEVCKRGYLRKQKHGHRRYFVLKLETADAPARLEYYENARKFRH      120
MUT_IRS4(p.G572W_fs*32)       GSRSDSESEEEDLPVGEEVCKRGYLRKQKHGHRRYFVLKLETADAPARLEYYENARKFRH      120
                              ************************************************************

WT_IRS4                       SVRAAAAAAAAAASGAAIPPLIPPRRVITLYQCFSVSQRADARYRHLIALFTQDEYFAMV      180
MUT_IRS4(p.G572W_fs*32)       SVRAAAAAAAAAASGAAIPPLIPPRRVITLYQCFSVSQRADARYRHLIALFTQDEYFAMV      180
                              ************************************************************

WT_IRS4                       AENESEQESWYLLLSRLILESKRRRCGTLGAQPDGEPAALAAAAAAEPPFYKDVWQVIVK      240
MUT_IRS4(p.G572W_fs*32)       AENESEQESWYLLLSRLILESKRRRCGTLGAQPDGEPAALAAAAAAEPPFYKDVWQVIVK      240
                              ************************************************************

WT_IRS4                       PRGLGHRKELSGVFRLCLTDEEVVFVRLNTEVASVVVQLLSIRRCGHSEQYFFLEVGRST      300
MUT_IRS4(p.G572W_fs*32)       PRGLGHRKELSGVFRLCLTDEEVVFVRLNTEVASVVVQLLSIRRCGHSEQYFFLEVGRST      300
                              ************************************************************

WT_IRS4                       VIGPGELWMQVDDCVVAQNMHELFLEKMRALCADEYRARCRSYSISIGAHLLTLLSARRH      360
MUT_IRS4(p.G572W_fs*32)       VIGPGELWMQVDDCVVAQNMHELFLEKMRALCADEYRARCRSYSISIGAHLLTLLSARRH      360
                              ************************************************************

WT_IRS4                       LGLVPLEPGGWLRRSRFEQFCHLRAIGDGEDEMLFTRRFVTPSEPVAHSRRGRLHLPRGR      420
MUT_IRS4(p.G572W_fs*32)       LGLVPLEPGGWLRRSRFEQFCHLRAIGDGEDEMLFTRRFVTPSEPVAHSRRGRLHLPRGR      420
                              ************************************************************

WT_IRS4                       RSRRAVSVPASFFRRLAPSPARPRHPAEAPNNGARLSSEVSGSGSGNFGEEGNPQGKEDQ      480
MUT_IRS4(p.G572W_fs*32)       RSRRAVSVPASFFRRLAPSPARPRHPAEAPNNGARLSSEVSGSGSGNFGEEGNPQGKEDQ      480
                              ************************************************************

WT_IRS4                       EGSGGDYMPMNNWGSGNGRGSGGGQGSNGQGSSSHSSGGNQCSGEGQGSRGGQGSNGQGS      540
MUT_IRS4(p.G572W_fs*32)       EGSGGDYMPMNNWGSGNGRGSGGGQGSNGQGSSSHSSGGNQCSGEGQGSRGGQGSNGQGS      540
                              ************************************************************

WT_IRS4                       GGNQCSRDGQGTAGGHGSGGGQRPGGGHGSGGGQGPGDGHGSGGGKNSGGGKGSGSGKGS      600
MUT_IRS4(p.G572W_fs*32)       GGNQCSRDGQGTAGGHGSGGGQRPGGGHGSGWWPGTWRWPWLRWWQELWGGQ--------      592
                              *******************************   *          ::  **:

WT_IRS4                       DGDGERGKSLKKRSYFGKLTQSKQQQMPPPPPPPPPPPPAGGTGGKGKSGGRFRLYFCVD      660
MUT_IRS4(p.G572W_fs*32)       -----RLRKWERIRW*--------------------------------------------      602
                                   * :. ::  :

WT_IRS4                       RGATKECKEAKEVKDAEIPEGAARGPHRARAFDEDEDDPYVPMRPGVATPLVSSSDYMPM      720
MUT_IRS4(p.G572W_fs*32)       ------------------------------------------------------------      602

WT_IRS4                       APQNVSASKKRHSRSPFEDSRGYMMMFPRVSPPPAPSPPKAPDTNKEDDSKDNDSESDYM      780
MUT_IRS4(p.G572W_fs*32)       ------------------------------------------------------------      602

WT_IRS4                       FMAPGAGAIPKNPRNPQGGSSSKSWSSYFSLPNPFRSSPLGQNDNSEYVPMLPGKFLGRG      840
MUT_IRS4(p.G572W_fs*32)       ------------------------------------------------------------      602

WT_IRS4                       LDKEVSYNWDPKDAASKPSGEGSFSKPGDGGSPSKPSDHEPPKNKAKRPNRLSFITKGYK      900
MUT_IRS4(p.G572W_fs*32)       ------------------------------------------------------------      602

WT_IRS4                       IKPKPQKPTHEQREADSSSDYVNMDFTKRESNTPAPSTQGLPDSWGIIAEPRQSAFSNYV      960
MUT_IRS4(p.G572W_fs*32)       ------------------------------------------------------------      602

WT_IRS4                       NVEFGVPFPNPANDLSDLLRAIPRANPLSLDSARWPLPPLPLSATGSNAIEEEGDYIEVI      1020
MUT_IRS4(p.G572W_fs*32)       ------------------------------------------------------------      602
```

Supplemental figure 1: Aligned IRS4 wildtype (WT) and mutated (p.G572W_fs*32) sequences. The alignment was performed using Clustal O (1.2.4) multiple sequence alignment tool (12).

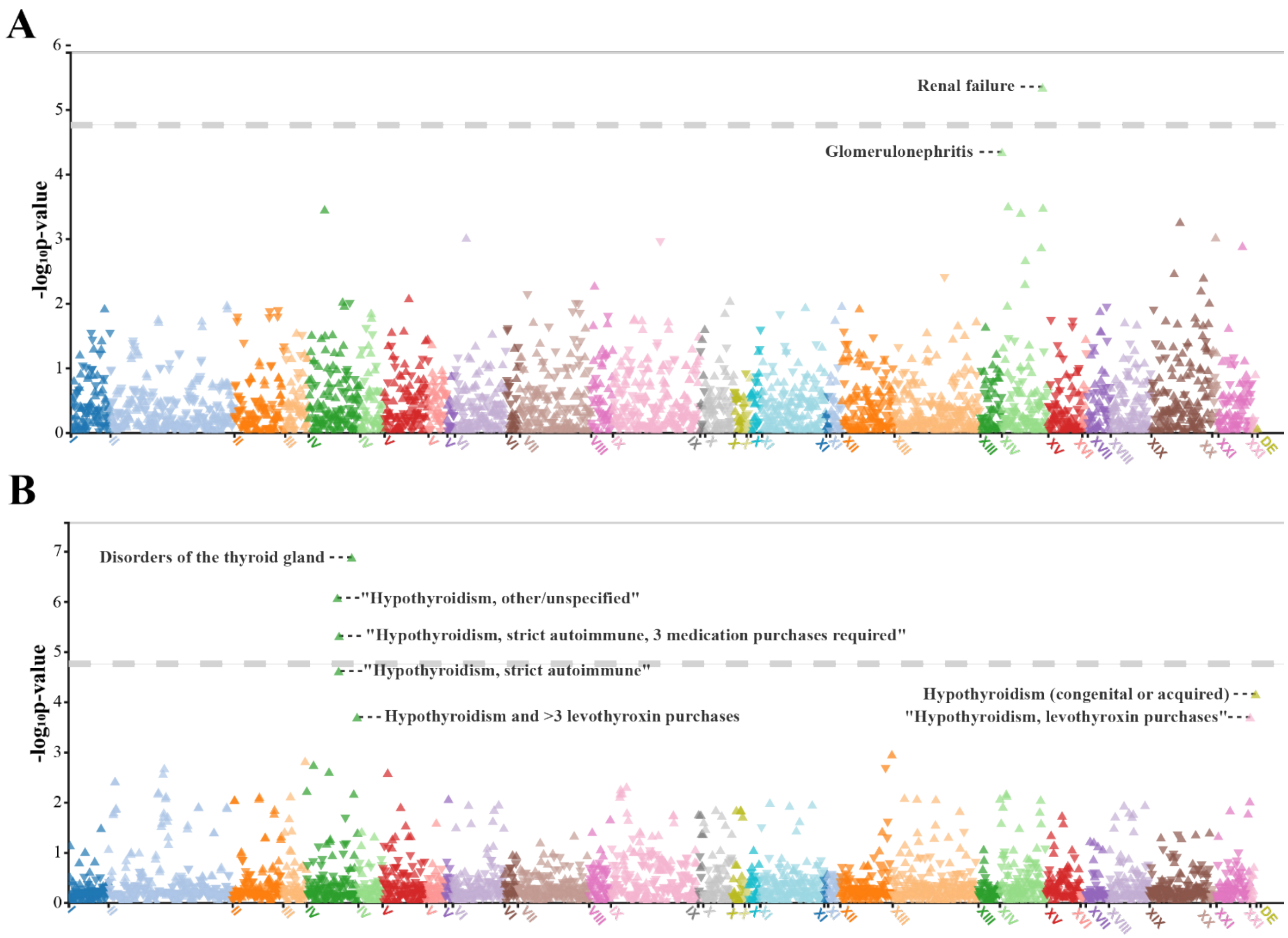

Supplemental figure 2: PheWeb images of the two IRS4 variants.
(A) rs1801164 showing significant association with renal failure and **(B)** rs1452561670, 20 kb downstream of IRS4 associated significantly with thyroid disorders and hypothyroidism over 2925 disease phenotypes in 13 endpoint categories as described in FinnGen (finngen.gitbook.io/documentation/).

Supplemental table 2

Primer name	Sequence (5’ > 3’)	Product size (bp)	$T_{annealing}$ (ºC)
A-IRS4ex1F	GAAACCAGTGTTCAGGCGAG	551	60
A-IRS4ex1R	TGGCACGTATGGGTCATCCT		
B-IRS4ex1F	GCTCCAGTAGCCATAGCTCG	755	
B-IRS4ex1R	AGGTGCTTTTGGAGGACTCG		

IRS4 primer sequences and annealing temperature used in PCR and Sanger-sequencing.

Data availability statement

The VCF-file of the clinical exome dataset from the index case presented in this study can be found in online repository.
